# Primary Hydatid Cyst of the Small Intestine: A Rare Case Report and Brief Review of the Literature

**DOI:** 10.7759/cureus.716

**Published:** 2016-07-28

**Authors:** Suleyman Caglar Ertekin, Tolga Ozmen

**Affiliations:** 1 General Surgery, Marmara University Hospital

**Keywords:** hydatid disease, small intestine, surgery, laparoscopy

## Abstract

Hydatid disease is an endemic disease especially in underdeveloped and developing countries affecting mostly the liver and lungs. The hydatid cysts located in other sites are mostly due to rupture of primary liver or splenic cysts. We present a primary small intestine hydatid cyst resected laparoscopically with the affected intestinal segment. As far as we know, this is the first report of a primary small intestine hydatid disease in the literature.

## Introduction

Hydatid cysts may be found in almost any site of the body, either from primary inoculation or via secondary spread. The liver (66%) and lung (25%) are the most commonly affected sites, but other organs (e.g. spleen, brain, muscle, kidneys, adrenal glands, bone, heart, pancreas) can also be affected [1-2]. Mesenteric hydatid disease can occur due to iatrogenic rupture of visceral (i.e., liver, spleen) hydatid cysts. Primary intestinal hydatid disease is exceptional.

Patients who have an extrahepatic hydatid cyst present mostly with abdominal pain and discomfort. Diagnosis can be challenging [3-4]. We present here an unusual case of primary intestinal hydatid cyst and a review of the literature.

## Case presentation

A 31-year-old woman presented with a palpable mass on the left upper quadrant of the abdomen. She had a 14-month history of intermittent abdominal pain attacks, discomfort, and early satiety. The patient's medical history and family history were unrevealing. Abdominal examination revealed a palpable, partially mobile mass at the left hypochondriac region.

**Figure 1:**
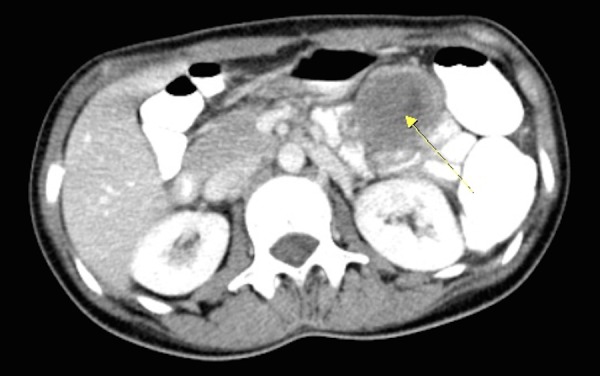
The Lesion (arrow) Appearing on Computed Tomography (CT)

**Figure 2:**
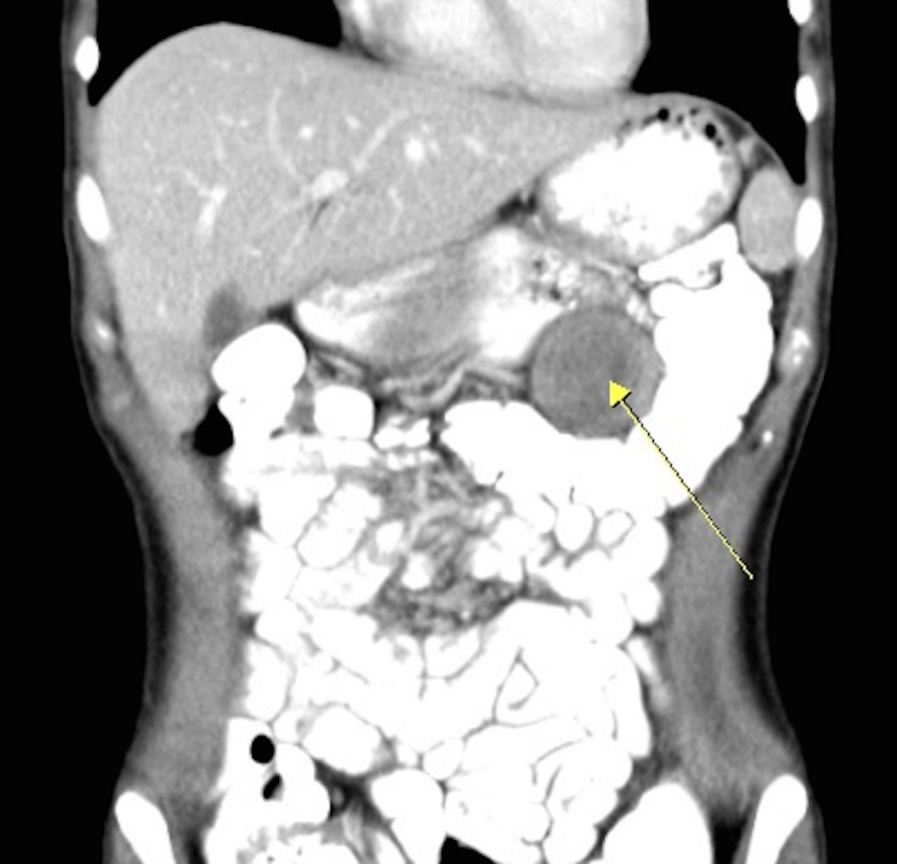
The Mass (arrow) in Coronal CT Plane

Laboratory findings were unremarkable. Hydatid serology (IHA) was negative. Abdominal computed tomography (CT) scans revealed a heterogeneous mass originated from the stomach wall, measuring 4.1 x 4.3 cm (Figures 1-2). No pathological appearance was detected in the chest x-ray. Upper GI endoscopy was also unrevealing.

A preliminary diagnosis of a gastrointestinal stromal tumor (GIST) originating from the stomach was made. During diagnostic laparoscopy, the stomach was intact, but we found a 5 cm mass located at the jejunum, 30 cm distally to the Treitz ligament. The affected jejunum segment was resected 5 cm proximally and 5 cm distally with a stapling device. The mass was taken out of the abdomen inside a specimen retrieval bag. The two jejunum ends were anastomosed. The postoperative period was uneventful and the patient was externalized on day three.

The pathology report revealed a 5.5 x 5 x 4 cm mass on the serosal aspect of the small intestine. The mass was rounded, had regular borders, and a white color. There was no association between the mucosa of the intestine and the mass. The inside cavity of the mass was filled with cheese-like necrotic tissue. There was no immune expression on CD117, DOG1, SMA, S100, and Ki-67 dying of the fibrotic capsule of the cystic lesion. Around the cystic lesion, periodic acid-Schiff (PAS) staining positive for cuticular membrane was detected. In the fibrotic tissue surrounding the cystic lesion, collagenation was detected with Masson's trichromatic dye. These histological and morphological findings supported a diagnosis of a hydatid cyst. After the pathological report of this rare clinical entity became absolute, informed consent was gained from the patient to publish these findings.

A three-cycle albendazole treatment was ordered to the patient. In each cycle, the patient took 400 mg albendazole twice a day for 28 days and stopped for 14 days. No recurrence was noted during the 12-month follow-up period.

## Discussion

Hydatid disease, caused by the *Echinococcus* *granulosus* is an endemic disease especially in regions like Eurasia and South American countries [5]. The small intestine is an unusual site for a hydatid cyst, and there are a few primary peritoneal hydatid cyst case reports in the literature [5]. The mechanism of infestation is not clear. For intestinal mesenteric hydatid cysts, dissemination via lymphatic or systemic circulation has been implicated as a possible route, and we think this might also be the mechanism in our case  [6].

Extrahepatic hydatid disease usually remains asymptomatic for years. Patients mostly present after the cyst becomes large enough to palpate or to cause non-specific symptoms as abdominal discomfort. The combination of clinical, laboratory, and radiological findings help for a preliminary diagnosis. Among all imaging modalities, ultrasonography is superior to both MRI and CT in visualization and evaluation of the morphology of liver cysts and hydatid disease [7]. In the literature, very high sensitivity (88% to 98%) and specificity (93% to 100%) rates are given for ultrasonography in the diagnosis of hydatid disease [7]. Abdominal cystic lesions (i.e., mesenteric cysts, ovarian cysts, lymphangioma) must be considered in the differential diagnosis [6]. In our routine practice, the workup of a patient with a mass located in the abdomen starts with a CT scan. Since the CT scan reported a non-cystic mass originating from the stomach wall, hydatid disease was not considered in the differential diagnosis, and we did not proceed with any other imaging modality.

Commonly used serological methods in the diagnosis of hydatid disease are the enzyme-linked immunosorbent assay (ELISA), the indirect hemagglutination test (IHA), the latex agglutination test and immunoblots [7]. Nevertheless, the usage of these tests still remains controversial due to inadequate sensitivity and specificity rates. In the literature, a wide range of sensitivity rates (50% to 100%) is given for the IHA test. The specificity rate is also reported to be 83% to 88% [7]. Studies suggest that combining the ELISA test and the IHA test increases the sensitivity up to 94.7% [8]. In our case, the IHA test was done in another clinic before admission to our hospital. Since it was negative and the imaging study was also not supporting a diagnosis of hydatid disease, no further serological test was done for this patient. There are some arguments given to explain false-negative IHA results. It is argued that only 60% to 80% of hydatid disease patients become seropositive. It is also argued that patients with a cystic lesion less than 9 cm diameter or a cystic lesion that is solitary, extrahepatic, unilocular, or degenerative are more prone to a false-negative IHA result [7]. Since in our case the lesion was 5 cm in diameter, solitary, and extrahepatic; these might be the reasons of serological false negativity.

The gold standard treatment for hydatid disease is complete surgical excision though according to the site of origin, partial or subtotal cystectomy can be performed to avoid adjacent organ injuries [9]. In our case we had a preliminary diagnosis of GIST, so we resected the affected intestinal segment and took the specimen out in a specimen retrieval bag. Mebendazole or albendazole is given to the patient adjuvantly to prevent recurrence [4]. In our case, we preferred albendazole treatment, and there was no recurrence in the 12-month follow-up period.

As far as our knowledge, this is the first case report of a primary small intestine hydatid cyst resected laparoscopically. 

## Conclusions

Hydatid disease is a significant public health problem in underdeveloped and developing countries affecting mostly the liver and lungs. Primary small intestine hydatid disease is a very rare clinical entity, which should be kept in mind for patients with an intra-abdominal mass.
